# Complete chloroplast genome sequence of *Parnassia brevistyla* (Celastraceae) and phylogenetic analysis with related species

**DOI:** 10.1080/23802359.2018.1524725

**Published:** 2018-10-26

**Authors:** Mingze Xia, Faqi Zhang, Hua Rao, Xiaofeng Chi, Gulzar Khan, Yu Zhang, Jingya Yu, Shilong Chen

**Affiliations:** a Key Laboratory of Adaptation and Evolution of Plateau Biota, Northwest Institute of Plateau Biology, Chinese Academy of Sciences, Xining, China;; b University of Chinese Academy of Sciences, Beijing, China;; c Qinghai Provincial Key Laboratory of Crop Molecular Breeding, Xining, China;; d College of Life Sciences, Shaanxi Normal University, Xi’an, China

**Keywords:** *Parnassia*, Celastraceae, chloroplast genome, Saxifragaceae, phylogenetic relationship

## Abstract

The taxonomic status of *Parnassia* has been widely discussed, which has been placed in more than five families, Parnassiaceae, Droseraceae, Saxifragaceae, and Celastraceae. Due to the lack of reliable genetic data, we sequenced and analyzed *P. brevistyla* chloroplast genome for future genetic study. The complete chloroplast genomes of *Parnassia brevistyla* was sequenced with NovaSeq 6000. The full length of *P. brevistyla* chloroplast genomes is 151,728 bp. A total of 114 unique genes, including 30 tRNA genes, four rRNA genes, and 80 protein-coding genes were found in the chloroplast genome. Using the whole chloroplast genome sequences alignment of 10 species from Celastraceae and Saxifragaceae, the phylogenetic relationship was built. The phylogenetic position of *P. brevistyla* was closely clustered with Celastraceae. The complete chloroplast genome of *P. brevistyla* provides utility information for further research of phylogenetic relationship and taxonomic status of *Parnassia*.

As a genus widely distributed in the Northern Hemisphere, most diverse in China and the Himalayas, the systematists have had some trouble in classifying *Parnassia* L. over the past century (Simmons 2004). The taxonomic status of *Parnassia* has been widely discussed, which has been placed in more than five families: Droseraceae (Pace 1912), Parnassiaceae (Takhtajan 1987), Saxifragaceae (Gu and Hultgård 2001), and Celastraceae (Simmons et al. 2001; Byng et al. 2016). However, most *Parnassia* studies still rely on a small amount of DNA fragments (Yang et al. 2012).

Fresh leaves of *P. brevistyla* (Voucher specimen accession No. Chen2013132; Geographic coordinates 31°10′N, 100°53′E) was sampled and quickly dried in silica gel. Voucher specimens were deposited into the Qinghai-Tibetan Plateau Museum of Biology (HNWP), Northwest Institute of Plateau Biology, Chinese Academy of Sciences. Total genomic DNA was extracted from approximately 10 mg of silica-dried leaf tissue by modification CTAB method (Doyle and Doyle 1987). After quantified and fragmented, DNA library preparation was performed following the protocol described in Thomson et al. (2018) and Chi et al. (2018). Libraries sequencing were carried out using NovaSeq 6000 (Illumina Inc., San Diego, CA, USA) with 150 bp paired-end reads. Totally 43,370,862 paired-end reads were obtained and 3,098,027 reads were assembled to the reference cp genome of *E. schensianus* (KY511610) (Wang et al. 2017). Raw reads were filtered in SOAPnuke Version1.3.0 to remove sequencing adaptors and low-quality bases (Chen et al. 2018). Clean reads were assembled with the programs SPAdes Version 3.10.1 (Bankevich et al. 2012), and aligned to the reference genome using BLAST v2.2.31 (http://blast.ncbi.nlm.nih.gov). The assemble accuracy around IR-LSC/SSC junctions had been tested by designing eight pairs of primers and amplify junction regions and the amplify result was consistent with the assemble result. Annotation was performed in CpGAVAS (Liu et al. 2012) coupled with manual adjustment of start/stop codons and intron/exon borders in SEQUIN Version 15.50 (https://www.ncbi.nlm.nih.gov/Sequin/) after BLAST searches. Part of tRNA genes was annotated by using tRNAscan-SE (Lowe and Chan 2016). The annotated chloroplast genome sequence was deposited into GenBank under the accession MG792145.

The complete genome size of *P. brevistyla* is 151,728 bp in length, containing the large single copy (LSC, 82,579), small single copy (SSC, 18,595) and two inverted repeats (IR, 25,277) regions. Overall GC contents of chloroplast genomes were 37.17%. A total of 114 unique genes, including 30 tRNA genes, four rRNA genes, and 80 protein-coding genes were found in four chloroplast genomes. In the phylogenetic tree, the position of *P. brevistyla* was closely clustered to Celastraceae with a high support rate, consistent with the results of the APG III system (Figure 1).

**Figure 1.**
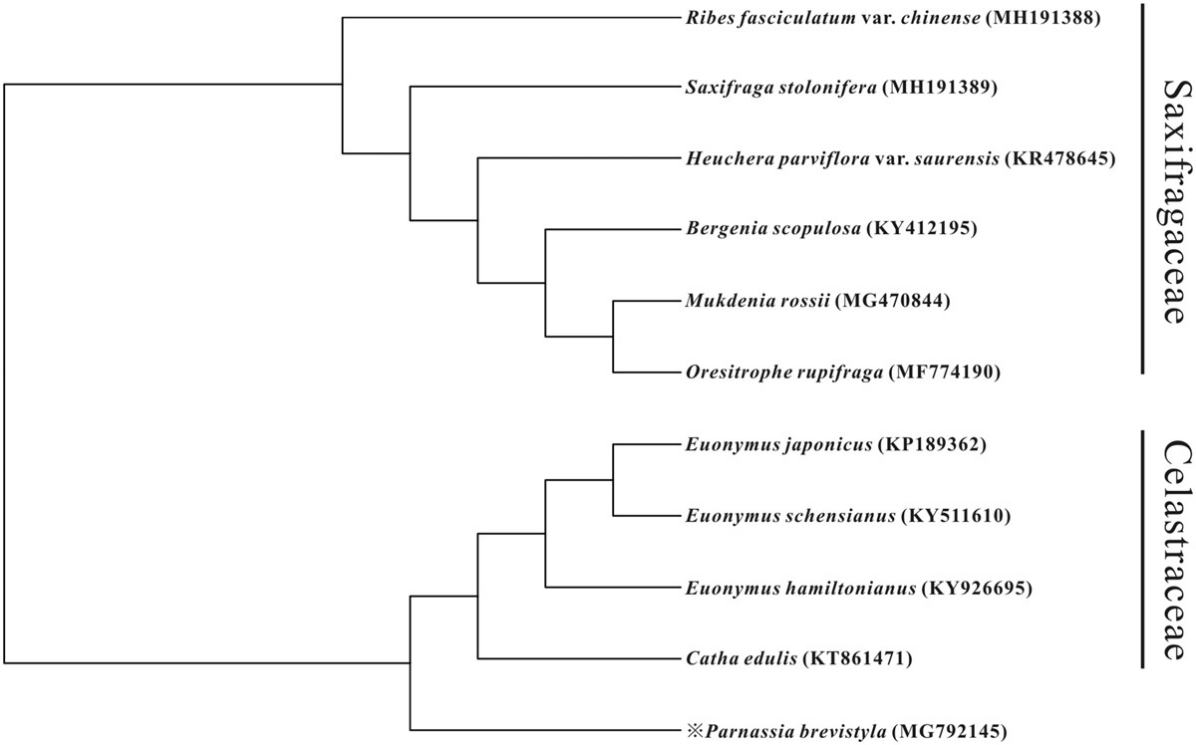
MP phylogenetic tree of *P. brevistyla* with 10 species in Celastraceae and Saxifragaceae was constructed by chloroplast genome sequences. All the branches were supported by 100% bootstrap values.
